# Comparing Immersive and Non-Immersive VR: Effects on Spatial Learning and Aesthetic Experience in Museum Settings

**DOI:** 10.3390/brainsci15080852

**Published:** 2025-08-11

**Authors:** Laura Piccardi, Marcello Massidda, Laura Travaglini, Sofia Pescarin, Marco Giancola, Massimiliano Palmiero, Matteo Deflorian, Sabrina Apollaro, Roberta Lista, Raffaella Nori

**Affiliations:** 1 Department of Psychology, Sapienza University of Rome, 00185 Rome, Italy; 2 IRCCS San Raffaele Cassino, 03043 Cassino, Italy; 3 Institute of Heritage Science, National Research Council, 50019 Sesto Fiorentino, Italylaura.travaglini@studio.unibo.it (L.T.); sofia.pescarin@cnr.it (S.P.); 4 Department of Biotechnological and Applied Clinical Sciences, University of L’Aquila, 67100 L’Aquila, Italy; marco.giancola@univaq.it; 5 Department of Communication Sciences, Università of Teramo, 64100 Teramo, Italy; mpalmiero@unite.it; 6 Department of Psychology, University of Bologna, 40127 Bologna, Italy; matteo.deflorian@studio.unibo.it (M.D.); sabrina.apollaro@studio.unibo.it (S.A.); roberta.lista2@studio.unibo.it (R.L.)

**Keywords:** spatial learning, aesthetic appreciation, virtual exploration, sense of immersion, computer-based environment, sociodemographic differences

## Abstract

Background/Objectives: The use of virtual reality (VR) solutions in design has rapidly increased globally. However, it remains unclear to what extent these technologies enhance people’s cognitive abilities. Understanding the impact of such technologies is essential for assessing their validity and effectiveness. In this controlled study, we investigated how HMD and non-immersive VR museum environments affect the ability to learn about the environment and the perception of the experience. Methods: A total of 87 college students (46 females) were randomly assigned to either HMD or non-immersive VR museum environments. Subsequently, they had to answer questions related to the sense of immersion and pleasantness of the museum experience, as well as their willingness to repeat similar museum experiences. Results: The results indicated that the HMD setting was preferred for its greater sense of immersion, pleasantness, and intention to repeat a similar experience. Conclusions: The data are discussed in the context of fostering appreciation and promoting the preservation of cultural heritage.

## 1. Introduction

The concept of a virtual museum was first proposed by André Malraux in 1947, who envisioned an imaginary museum that transcended physical boundaries, allowing visitors to experience art and culture from anywhere in the world, free from walls and limitations. Today, a virtual museum can be understood as a digital collection encompassing images, audio, texts, and other data of historical, scientific, or cultural significance. The integration of virtual reality (VR) technology and digital twins (i.e., highly detailed virtual replicas of physical spaces, objects, or processes, developed using real-world data and 3D modeling) in museum exhibitions has proven to be a groundbreaking development in cultural heritage preservation and public engagement [1,2].

This technological revolution has transformed how visitors interact with art and historical narratives, offering immersive experiences that go beyond the limitations of traditional display methods [3]. The importance of VR in museum contexts extends beyond mere technological novelty. VR allows visitors to explore museum spaces in ways previously impossible, enabling them to experience reconstructed historical environments, interact with fragile objects without physical contact, and access collections that may be geographically or temporally distant [4,5]. Digital twin technology further enhances this capability by creating precise digital replicas of museums, exhibitions, and individual objects, providing unprecedented opportunities for preservation, analysis, and virtual accessibility [6].

A recent bibliometric analysis examining 722 publications revealed that immersive technology applications in museum exhibitions have grown exponentially, with VR being the dominant technology for enhancing visitor experiences [7].

The COVID-19 pandemic acted as a pivotal force in accelerating the adoption of digital technologies within the museum sector. Agostino et al. [8] revealed that museums around the globe swiftly transitioned to digital platforms, making virtual exhibitions vital for sustaining cultural engagement during lockdowns. Museums worldwide rapidly developed virtual exhibitions, 360-degree tours, and VR experiences to maintain public engagement during lockdowns [8,9]. Chen et al. [10] examined users’ acceptance of online virtual reality museum exhibitions, finding that technological factors significantly influence visitor engagement, with perceived usefulness and ease of use being primary determinants of acceptance.

Lee et al. [11] and Tussyadiah et al. [12] highlight that a highly immersive experience in a virtual museum significantly increases participants’ future intentions to attend live exhibitions. The authors emphasize that their research on VR experiences in tourism provides a better conceptualization of technology’s role, illustrating how VR can transform users’ attitudes towards actual consumption, specifically, traveling to various museums and exploring cultural heritage sites. This phenomenon is supported by the theory that immersive VR effectively alters visitors’ attitudes and perceptions toward destinations, fostering more positive outlooks on locations. For this reason, VR has been increasingly utilized in cultural tourism, as its features significantly contribute to the tourism industry’s objective of providing unique and enhanced experiences for travelers [11,13]. This technology facilitates multi-sensory learning experiences, allowing visitors to virtually walk through ancient ruins, manipulate historical objects, and witness artistic processes in real-time simulations. Wang et al. [14] demonstrated that well-designed VR systems significantly improve visitors’ satisfaction and learning outcomes compared to traditional exhibition methods. Post-pandemic museum strategies have increasingly focused on dialogical engagement models that blend physical and digital experiences [15]. As these technologies mature, they offer unprecedented opportunities for cultural institutions to fulfill their educational missions while reaching broader, more diverse audiences than ever before.

Furthermore, this technological evolution, combined with increased competitiveness in the marketplace, has led to lower costs, thus facilitating the adoption of VR by both consumers and professionals (e.g., [11]).

VR environments exist across a spectrum of immersive technologies, each offering different levels of sensory engagement and user experience. Indeed, both head-mounted displays (HMD) like Oculus devices and desktop-based VR, i.e., non-immersive VR systems, create VR environments, but their distinct degrees of immersion produce measurably different effects on users (e.g., [16]).

Specifically, HMD-VR can engage multiple senses, such as sight and hearing, and, in some cases, touch, providing a multisensory experience. Conversely, the non-immersive VR experience is primarily visual and auditory, with more limited sensory involvement. Additionally, with HMD-VR, the participant feels a deep sense of immersion, and the feeling of “being there” is not merely a mechanical outcome; it emerges from a complex interplay of context, individual differences, and expectations [17]. Cummings and Bailenson’s [18] meta-analysis highlights that higher levels of immersion are generally associated with greater presence but also emphasizes that specific features (e.g., field of view and head tracking) play a critical role in modulating these effects.

Consequently, researchers have attempted to identify the most essential characteristics of interfaces that can effectively evoke this psychological state in simulated environments. According to Riva and Valataro [19], the cognitive-sensory mechanism of presence is activated when the “self” undergoes a deep sense of immersion in a given situation. The strong link between engagement and immersion is so pronounced that some studies do not adequately distinguish between the two [20]. As noted by Mütterlein [21], immersion, viewed as a psychological state, can be assessed through the interplay of achieving a flow state characterized by deep concentration and focus and another characterized by engrossing focus where one loses track of time. Among these states, flow, as described by Csíkszentmihályi et al. [22,23], is particularly aligned with the experience of immersion. According to Csíkszentmihályi [24], flow represents a state of mind of full absorption, where action and consciousness merge. Factors such as vividness, focused immersion, curiosity, and control during VR experiences significantly enhance the sense of presence (i.e., the degree to which an individual feels immersed in the virtual environment, [25]), facilitating the flow state [26].

HMD-VR exerts a profound influence on cognitive, behavioral, and individual differences when compared to non-immersive VR. Both types of VR offer a degree of immersion and can induce a sense of presence in users. While the non-immersive VR is constrained by its technological limitations (i.e., being primarily tied to monitors, mice, and other input devices), it represents a cost-effective and viable option for engaging with VR applications [23,24]. Research demonstrates that HMD-VR can elicit stronger behavioral responses and inspire greater intentions to act.

HMD-VR is associated with both stronger emotional responses and an overall improved user experience [27]. The heightened sense of presence in HMD-VR is attributed to its stereoscopic vision that responds to the user’s head position, allowing for more natural movement within the environment and precise tracking of the participant’s actions [28]. Nevertheless, there is a limited number of studies that specifically explore the differences between non-immersive virtual reality (desktop-based VR) and immersive virtual reality (HMD-VR), particularly in terms of their applications and interface usage [11], which show contrasting results. For example, Srivastava et al. [29] revealed that non-immersive VR led to better spatial recall (e.g., map drawing), less motion sickness, lower workload, and greater usability than HMD-VR when physical movement was restricted, attributing poorer spatial learning in HMD-VR to a lack of idiothetic cues. Conversely, Cadet and Chainay [30] revealed that HMD-VR did not directly enhance memory performance but the sense of presence was increased by both device types. Moreover, several neuropsychological studies have compared visual attention and working memory in HMD-VR vs. non-immersive VR. Li et al. [31] found enhanced selective attention in HMD environments, while other experiments (e.g., visual n-back and vigilance tasks) found no significant performance differences between HMD-VR and non-immersive VR [32,33,34]. Table 1 reports the search domains and the principal results regarding HMD-VR and non-immersive VR comparison.

Users’ experiences may also be affected by other factors related to individual differences. For instance, gender differences in user experience and performance with HMDs and non-immersive VR have been studied, with a focus on simulator sickness, immersion, cognitive performance, and motor skill transfer [39,40,41,42]. Generally speaking, women are more likely to experience simulator sickness in HMD-VR, but gender differences in immersion, presence, and performance are inconsistent and often depend on the specific VR context and task. In addition to gender, age and prior experience are also especially important, with older adults and those less familiar with technology often performing better with non-immersive VR, while younger or more experienced users adapt more easily to HMD-VR (e.g., [42,43]).

To investigate differences between HMD-VR and non-immersive VR, we used the same virtual environment, a digital twin of a real exhibition, titled “L’altro Renaissance—Ulisse Aldrovandi and the Wonders of the World,” held at Palazzo Poggi in Bologna (Italy) from December 2022 to May 2023. The exhibition centered around a common theme, with several artifacts varying greatly in their geometric shapes and surface textures. While previous studies have focused on presence, usability, and enjoyment, few have combined cognitive measures (i.e., spatial learning performance) with affective and behavioral outcomes in a naturalistic, culturally significant context like a real museum exhibition. Our inclusion of the digital twin and the comparison between HMD-VR and non-immersive VR for both cognitive and motivational outcomes (e.g., willingness to return to similar experiences) offers a unique contribution. To understand the relationship between sociodemographic factors and cultural interests and their influence on enjoyment of the experience, environmental immersion, the inclination to revisit the museum, and learning time, we controlled for these elements. After controlling for these factors, we hypothesized that H1: the HMD experience would be perceived as more pleasurable and immersive, leading to a stronger desire to repeat it than the non-immersive VR experience. Furthermore, we also hypothesized that H2: the type of immersive environment, HMD or non-immersive VR, would influence the time participants required to learn the environment. Specifically, participants using HMD-VR would take less time to learn the environment. This expectation arose from the sense of presence [18], which is grounded in embodied cognition theory, and fostering enhanced visual attention orientation and improved visual perception in virtual environments. Furthermore, this study examined whether flow (captured in terms of pleasantness and perceived immersion) mediated the association between HMD-VR and non-immersive VR and learning time. Accordingly, H3 stated that the more the individual was exposed to an immersive condition (i.e., HMD-VR), the greater the flow experienced, which, in turn, would negatively affect learning time.

## 2. Materials and Methods

### 2.1. Participants

Participants were recruited through social media posts and word of mouth, using a snowball method for data gathering [44], yielding a total of 96 individuals. Nine participants were excluded due to previous brain injuries and unfamiliarity with the virtual environment. Consequently, the final sample comprised 87 participants (38 males), who were randomly assigned to either the HMD-VR group (17 males; M age = 22.94 years SD = 0.68 and 27 females; M age =23.26 years SD = 0.54) or the non-immersive VR group (21 males; M age = 23.05 years SD = 0.61 and 22 females; M age = 23.68 years SD = 0.60). The mean age of the sample was 23.25 (range: 18 to 34 years, SD = 2.78 years), and the mean years of education was 15.08 (SD = 2.32 years). To ensure no differences emerged between the two groups, we performed ANOVAs considering interest in cultural heritage and frequency of PC use (see Section 2). No differences were found between the HMD and non-immersive VR groups (interest in cultural heritage, F (1, 86) = 0.49, *p* = 0.49, HMD M = 2.94; SD = 0.80, non-immersive VR M = 2.81 SD = 0,77; frequency of PC use: F (1, 86) = 0.22, *p* = 0.64, HMD M = 3.59; SD = 3.34 h, non-immersive VR M = 3.91; SD = 2.99 h). No participants reported cybersickness.

The research adhered to the ethical principles outlined in the Declaration of Helsinki. Ethical approval for this study was obtained from the Institutional Review Board of the University of Bologna (Italy). Written informed consent was obtained from all the participants before their involvement in the study (IRB of the University of Bologna, Italy; Protocol number: 8598).

### 2.2. Measures

At the beginning, participants were asked to complete the following questionnaires:

Anamnesis questionnaire [45]. Participants answered questions about early spatial orientation issues, major psychiatric illnesses, traumatic brain injury, learning disabilities, and substance abuse. For neurological aspects, they specified experiences with head trauma, ischemic attacks, encephalitis, infections, and complications. Regarding psychiatric history, they reported on depression, anxiety, psychosis, OCD, eating disorders, PTSD, schizophrenia, and phobias, including treatment history. Substance use inquiries covered alcohol frequency and drug use (e.g., cannabis, amphetamines, and cocaine), detailing usage timing and frequency.

The Computer and Video Game Use Questionnaire (CUQ: modified from [46,47]) includes five questions designed to measure daily usage patterns. This questionnaire features two items that employ a Likert scale, ranging from 1 (“Every day”) to 5 (“A few times a year or less”), to assess the frequency of computer and video game engagement. Additionally, the remaining questions request an estimate of daily hours spent playing, specifically for those who select “Every day,” and also examine whether the game character navigates within a 3D space.

The Scale for the Assessment of Caring for Cultural Heritage (CHARE [48]) includes 16 items requiring an answer on a Likert scale (from 1—“never” to 6—“always”). These items investigate the frequency with which individuals engage in positive interactions or reflections concerning cultural heritage. They are categorized into behavioral classes, arranged in a proposed sequence of increasing difficulty.

**Figure 1:**
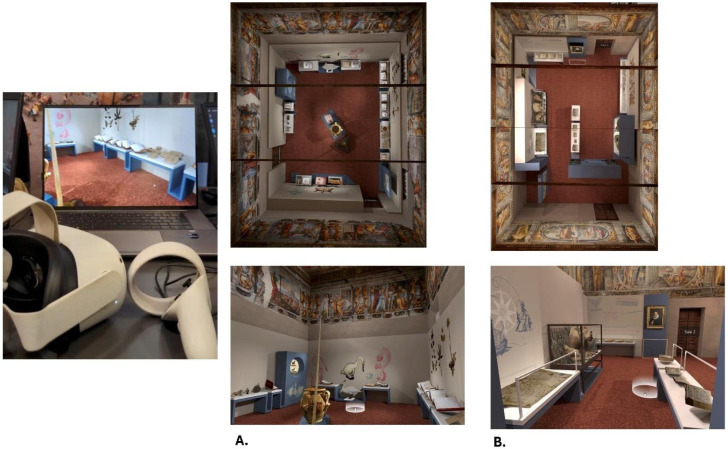
The two rooms are shown in map view and first-person perspectives. (**A**). The first room; (**B**). the second room.

**Figure 2:**
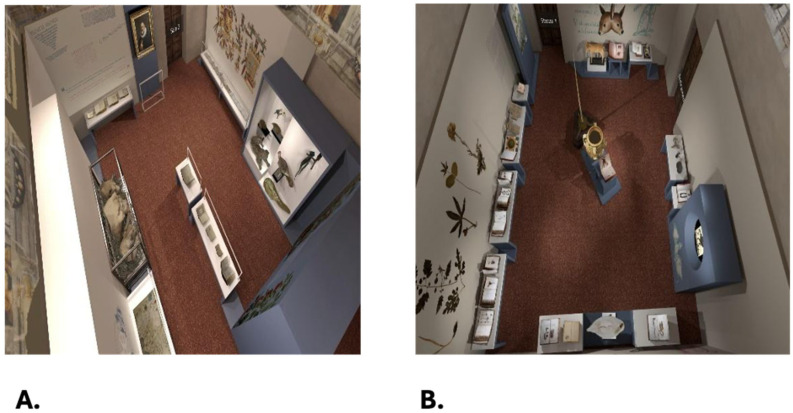
Object distribution in the museum rooms. (**A**). Room 1; (**B**). Room 2.

Museum environment: Participants explored a virtual environment, specifically a “digital replica” or digital twin of a real exhibition, titled “L’altro Renaissance—Ulisse Aldrovandi and the Wonders of the World” (https://site.unibo.it/aldrovandi500/en/mostra-l-altro-rinascimento (accessed on 1 January 2025) held at Palazzo Poggi (Via Zamboni 33) in Bologna, Italy, from 8 December 2022 to 28 May 2023. The digital twin used for the experiment is part of an ongoing project and one of the objectives (Spoke 4) of Project CHANGES. The experiment focused on the first two rooms of the digital twin (see Figure 1). Room 1 contained 22 artworks and Room 2 contained 25, including books, animal models, and manuscripts. The digital rooms also included graphic and lighting effects to create a photorealistic experience. Figure 2 shows the distribution and style of the rooms involved in the experiment. Although the exhibition centered around a common theme, the showcased objects varied greatly in their geometric shapes and surface textures. Participants could identify each object simply by hovering their cursor over it.

Moreover, it was necessary to record the spatial learning time of each participant and to maintain direct and real-time control over the participants’ museum visits. For this purpose, an ad hoc application, using ATON software (https://osiris.itabc.cnr.it/aton/ (accessed on 1 January 2024), [49]), was created and previously tested [28], allowing an admin user (the experimenter) to control the regular user’s session (participant). The interface of the admin panel was specifically designed to provide experimenters with a straightforward step-by-step wizard, facilitating the configuration of the session and the conducting of the experiment. This design ensured that the tool was user-friendly and efficient for psychologists to use during experimental sessions. Therefore, while the participant navigated the museum environment through one device (user), the experimenter was able to observe them through a second device (controller). While the controller was always a laptop computer, the user device depended on the experimental condition; it could be a second laptop computer (non-immersive VR) or an HMD-VR, the Oculus Quest 2. The application, installed on the controller computer, allowed for the creation of a local connection with the second device through an LAN network.

### 2.3. Procedure

Participants were divided into two experimental groups and randomly assigned to one of two conditions, “non-immersive VR,” in which participants interacted with the environment through mouse and keyboard, or “HMD-VR”, using the Oculus Quest 2’s head-mounted display and controller for an immersive experience. Each participant engaged in the experiment by initially completing questionnaires, then exploring a virtual environment, and finally completing a spatial task that allowed for assessment of the spatial learning of the environment. To avoid order and presentation effects of the material, the order of completing the questionnaires and the virtual museum experience was counterbalanced among participants.

**Figure 3:**
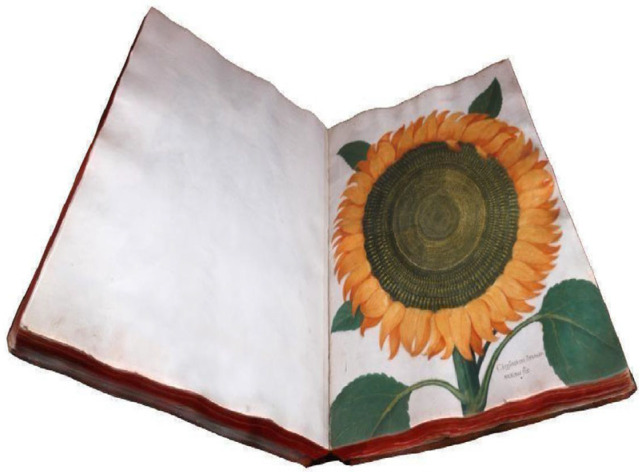
A book with a digital sunflower displayed at the Ulisse Aldrovandi exhibition that participants were asked to recognize and locate within the digital twin exhibition.

Before beginning the virtual exploration, participants were trained on how to navigate around the virtual museum and informed that there were two rooms. The experimental session started with the experimenter positioning the participant at the threshold of the first room. Participants could move freely from room to room within the exhibit, and no time limit was imposed. Unlike non-immersive VR participants, those in the HMD-VR group could experience a heightened sense of presence by walking naturally within the spatial confines defined by the boundaries of the Space and Virtual Reality Lab (14 × 10 m; https://spaceandvirtualrealitylab.com/) during the experiment. When participants believed they had sufficiently explored and understood the environment, they would inform the experimenter. The experimenter stopped the exploration and returned the participant to the first room, showed them an object (see Figure 3), and asked whether it was present in the explored environment.

When a correct response was given, the participant would be instructed to locate it once more as quickly as possible to verify that memory was involved and not a lucky guess. If the participants gave a wrong answer or could not locate the object, they were directed to go back to the museum and continue their exploration until they were more comfortable navigating the space.

At the participant’s second signal, the researcher would repeat the earlier process, presenting a different object of comparable difficulty. This procedure ensured that participants had familiarized themselves with the museum environment. Among the participants, only 10, 5 in the HMD condition and 5 in the non-immersive VR condition, incorrectly identified the first requested object and were required to repeat the learning phase within the museum environment. Subsequently, they had to recognize the second target item. All 10 participants, after this second attempt, had learned the environment. Therefore, the total learning time was calculated by summing the durations of both learning sessions. Once participants had explored the museum environment under one of the two conditions, they were asked three questions: one about the enjoyment of the experience (“How much did you enjoy exploring the museum?”), one about the sense of immersion (“How immersive did you find exploring the museum?”), and one about the intention to repeat a similar experience (“How much would you like to repeat a similar museum experience?”). The three questions were presented in randomized order to all participants, who had to answer them on a scale from 0 (not at all) to 100 (very much). The procedure lasted approximately 45 min.

## 3. Statistical Analysis

Data analysis was performed using IBM SPSS Statistics Package 28. Descriptive statistics were calculated for all variables to evaluate central tendencies and variability. Initially, multiple regression analyses were conducted to determine whether sociodemographic variables predicted how participants experienced the exhibition. Subsequently, we examined the influence of various proposed models on reported levels of immersion, overall enjoyment, and the likelihood of participants seeking similar experiences in the future. Finally, the contribution of each variable to the overall model was analyzed. A one-way ANOVA with group (HMD vs. non-immersive VR) as the independent variable and time (minutes) spent exploring the virtual exhibition was performed. The analyses were performed with alpha set to 0.05. The ANOVA was one-tailed. To examine the mediating effects of pleasantness and perceived immersion in the relationship between VR condition (HMD vs. non-immersive VR) and learning time, a mediation analysis was conducted using Hayes’ PROCESS Macro for SPSS version 3.5 [50]. The indirect effects were estimated using a bootstrap resampling procedure with 5000 iterations and 95% bias-corrected confidence intervals. Bootstrapping is a non-parametric approach that provides robust estimates of mediating effects even in small samples with *n* < 100 [51].

### 3.1. Results

To control for spurious effects due to sociodemographic variables, we performed a series of correlation analyses among gender, age, level of education, frequency of computer use assessed with the CUQ, and interest in cultural heritage through CHARE, and the following measures: perceived immersion, pleasantness, intention to repeat similar experiences, and learning time. Table 2 presents these correlation results.

**Figure 4:**
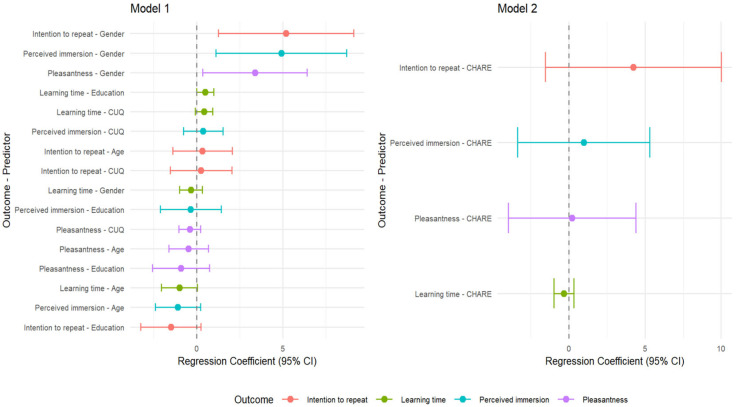
Dot and whisker plots for both regression models (Model 1 and Model 2).

Moreover, we performed four multiple linear regression analyses considering gender, age, level of education, and the CUQ (Model 1) and CHARE (Model 2) as independent variables, with the following dependent variables: pleasantness, perceived immersion, intention to repeat similar experiences, and learning time. Gender coded with men as −1 and women as 1, as suggested by Howitt and Cramer [52]. None of the models significantly predicted pleasantness (Model 1, F (4, 82) = 1.61, adj r^2^ = 0.02, *p* = 0.18; age, *p* = 0.49, 95% CI: −1.63–0.67 gender, *p* = 0.05, 95% CI: 0.36–6.40; level of education, *p* = 0.35, 95% CI: −2.58–0.73; CUQ, *p* = 0.97, 95% CI: −1.031–0.22; Model 2, F (1, 81) = 1.28, adj r^2^ = 0.02, *p* = 0.28; CHARE, *p* = 0.86, 95% CI: −3.98–4.39). Similarly, none of the models significantly predicted perceived immersion (Model 1, F (4, 82) = 2.19, adj r^2^ = 0.05, *p* = 0.08; age, *p* = 0.19 95% CI: −2.39–0.21; gender, *p* = 0.01, 95% CI: 1.12–8.705; level of education, *p* = 0.89, 95% CI: −2.11–1.42; CUQ, *p* = 0.52, 95% CI: −0.78–1.52; Model 2, F (1, 81) = 1.83, adj r^2^ = 0.05, *p* = 0.12; CHARE, *p* = 0.52, 95% CI: −3.37–5.32). No significant prediction was found for the intention to repeat similar experiences (Model 1, F (4, 82) = 1.97, adj r^2^ = 0.04, *p* = 0.11; age, *p* = 0.61, 95% CI: −1.40–2.06; gender, *p* = 0.01, 95% CI: 1.26–9.12; level of education, *p* = 0.19, 95% CI: −3.24–0.24; CUQ, *p* = 0.83, 95% CI: −1.53–1122.04; Model 2, F (1, 81) = 1.83, adj r^2^ = 0.05, *p* = 0.12; CHARE, *p* = 0,27, 95% CI: −1.56–10.02). Finally, no significant prediction was found for the learning time (Model 1, F (4, 82) = 2.00, adj r^2^ = 0.04, *p* = 0.10; age, *p* = 0.05, 95%, CI: −2.05–0.04; gender, *p* = 0.35, 95% CI: −0.99–0.33; level of education, *p* = 0.96, 95% CI: −0.01–1.00; CUQ, *p* = 0.97, 95% CI: −0.08–0.93; Model 2, F (1, 81) = 1.60, adj r^2^ = 0.03, *p* = 0.17; CHARE, *p* = 0.74, 95% CI: −0.98–0.33). Figure 4 presents the dot and whisker plots for both regression models (Model 1 and Model 2).

#### 3.1.1. H1: Influence of Different Levels of Immersion on Perceived Immersion, Pleasantness, and Intention to Repeat Similar Experiences

**Figure 5:**
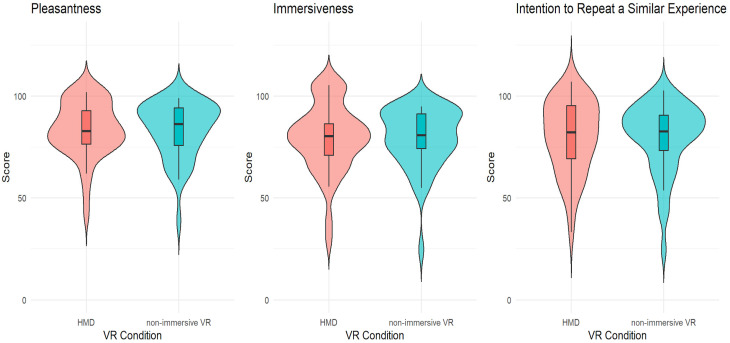
Violin plots displaying the distribution of pleasantness, immersion, and intention to repeat similar experiences across the VR conditions.

Based on correlation analysis, we performed an ANCOVA analysis considering the VR condition (HMD vs. non-immersive VR) as independent variables and gender as a covariate, with perceived immersion as the dependent variable. The HMD condition differed significantly from the non-immersive VR, demonstrating higher perceived immersion scores (F (1, 86) = 7.76, *p* = 0.01, *η*2 = 0.09). Descriptive statistics are reported in Figure 5. Additionally, there was also a significant effect of gender as a covariate. Perceived immersion was higher in women than in men: F (1, 86) = 4.23, *p* = 0.043, *η*2 = 0.05.

We performed an ANOVA to investigate the effects of exploration in HMD and non-immersive VR on pleasantness. Results revealed that pleasantness during virtual museum experience increased significantly with HMD, F (1, 86) = 5.49, *p* = 0.02, *η*2 = 0.06 (see Figure 5).

Finally, based on correlation analysis, we performed an ANCOVA using gender and cultural heritage interest as covariates to investigate the effects of VR condition (HMD vs. non-immersive VR) on the intention to repeat a similar experience. Results revealed that the intention to repeat a similar experience was significantly higher in the HMD condition: F (1, 86) = 5.13, *p* = 0.03, *η*2 = 0.06 (see Figure 5).

#### 3.1.2. H2. HMD vs. Non-Immersive VR Experience Affects the Time Participants Require to Learn the Museum Environment

Based on correlation analysis, we performed an ANCOVA to examine the impact of the HMD vs. non-immersive VR conditions, using age as a covariate, on learning time within the virtual exhibition, as well as its relation to reenactment ability as assessed by spatial tasks. Interestingly, the experimental condition did not yield significant results on learning time (F (1, 86) = 0.397, *p* = 0.530). Regardless of whether participants engaged with HMD or with non-immersive VR, their learning time remained consistent (HMD, M = 6.60 min, SD = 0.48; non-immersive VR, M = 6.12 min, SD = 0.47).

#### 3.1.3. H3. The More the Individual Is Exposed to an Immersive Condition, the Greater the Flow Experienced, Which, in Turn, Would Negatively Affect Learning Time

The mediation analysis revealed that, after controlling for age, gender, education, frequency of PC use, and CHARE, neither pleasantness (*B* = 0.39, *SE* = 0.28, 95% CI [–0.016, 1.07]) nor perceived immersion (*B* = –0.11, *SE* = 0.26, 95% CI [–0.69, 0.37]) mediated the relationship between VR condition and learning time. Additionally, the direct effect of VR condition on learning time was also not significant (*B* = 0.23, *SE* = 0.69, 95% CI [–1.15, 1.61]) nor was the total effect (*B* = 0.52, *SE* = 0.67, 95% CI [–0.81, 1.84]). The *R^2^* for the entire model was 0.11. Table 3 reports all paths of the mediation analysis.

## 4. Discussion

This study is grounded in the theoretical framework of the technological revolution’s impact on the enjoyment of art. Our findings show that technological immersion influences virtual cultural experiences, which is important for museum practices and VR research. Our results showed that the level of immersion (HMD vs. non-immersive VR) is important for determining aesthetic appreciation as well as willingness to re-experience a similar exhibition. Among the sociodemographic factors analyzed, we found that gender and people’s interest in cultural heritage correlate with virtual museum experiences. Women showed higher perceived immersion with virtual cultural heritage content than men; additionally, women and people with a high interest in cultural heritage are more prone to repeat the virtual museum experience. Pallavicini et al. [53] suggested that women during VR experiences activate more brain areas involved in emotional processing than men, and this could explain why women in our study reported significantly higher immersion scores across both HMD and non-immersive VR conditions. Additionally, people who have an interest in cultural heritage are likely to want to repeat the experience regardless of how it is presented, due to their subject interest rather than presentation mode. Furthermore, we found that participants in the HMD condition reported higher levels of immersion, greater enjoyment, and stronger intentions to participate in similar virtual museum experiences in the future. This study aligns with research demonstrating how the clarity and vividness of VR imagery play a crucial role in enhancing users’ sense of presence. During the HMD experience, individuals tend to become deeply engaged, effectively blocking out external distractions (focused immersion), while also experiencing enhanced sense of control over the virtual exploration (perceived control) and increased experiential pleasure, probably also due to the clarity and accuracy of object detail (e.g., [54,55]). While our work shows differences in metacognitive aspects, it found no differences between HMD and non-immersive VR in cognitive aspects, specifically in environmental learning. Our study is not the only one that has failed to find differences between HMD and non-immersive VR in cognitive tasks. For instance, O’Toole et al. [56] found no significant difference in performance between HMD and traditional non-immersive VR conditions for a musical pitch–color association test, suggesting that, for this specific auditory–visual task, the display type did not influence results. Similarly, in a cultural serious game designed to teach communication skills, no performance difference was found between participants using non-immersive VR and HMD-VR versions, indicating similar learning outcomes across platforms (for a review, see [57]). Similar findings emerged in aviation safety training; improvements in knowledge gain and self-efficacy were maintained regardless of whether participants used non-immersive VR or HMDs, though engagement and presence were higher with HMDs [58]. The absence of significant differences between HMD and desktop systems may be related to the task type participants perform within the environments. The easier the task, the fewer differences emerge between HMD and desktop systems. Consistent with this, studies by Feng et al. [59,60] examined pedestrian wayfinding and route choice in multi-level buildings. The authors found that, while non-immersive VR users performed better on wayfinding tasks, route, and exit choices, the overall user experience was similar between non-immersive VR and HMD groups, suggesting that, for simple tasks, both platforms yield comparable behavioral outcomes. Our study involved a very simple task: learning the museum environment and recognizing and localizing one object within it. Plechatà et al. [49] showed that young adults’ performance on memory tasks investigating recall ability remained stable regardless of whether non-immersive VR or HMD was used, though seniors performed better with non-immersive VR. Our sample consisted of young people, and this could explain the absence of differences between HMD and non-immersive VR. We found a correlation between age and learning time, even though the oldest participants were only 35 years old. In terms of computer experience, there were no differences between the two groups. This suggests that, with technological devices, even small age differences may affect learning time, perhaps because younger people tend to play video games and thus are more accustomed to three-dimensional environments, regardless of presentation mode (HMD vs. non-immersive VR), leading to shorter learning time.

In summary, our findings suggest that cultural institutions can significantly enhance visitor engagement by investing in VR technology. Cost need not be prohibitive; what matters is focusing on innovations that enrich the experience, thereby encouraging individuals to return and revisit exhibitions. Our findings align with Lee et al. [11] and Tussyadiah et al. [12], who found that immersive VR experiences boost visitors’ intentions to attend live exhibitions. HMD-VR users are more likely to repeat virtual experiences, indicating that immersive virtual museum visits can effectively promote physical cultural institutions and support cultural tourism and heritage engagement. However, it is important to acknowledge that intention does not always translate into actual behavior. As highlighted by Ajzen’s Theory of Planned Behavior [61,62,63], while intention is a strong predictor of action, it is influenced by several mediating factors such as perceived behavioral control, situational constraints, and habitual tendencies. Therefore, although our findings indicate that immersive VR can enhance interest in live cultural experiences, further longitudinal research is needed to verify whether these intentions lead to concrete visits to museums or heritage sites. This distinction is crucial for assessing the real-world impact of virtual museum initiatives on cultural tourism and public engagement.

Contrary to our expectations, we found no significant differences between HMD-VR and non-immersive VR conditions in learning time. Participants took similar amounts of time to learn the virtual environment, regardless of the interface. While HMDs provided superior experiential outcomes, this did not translate into faster environmental learning. Several factors may explain this unexpected result. First, the virtual environment may have been sufficiently well-designed that both interface types provided adequate spatial information for navigation. Second, the novelty of the VR experience itself, regardless of the interface type, may have created a leveling effect where participants needed similar amounts of time to adapt to the virtual interaction paradigm. Percentual realism in both HDM and non-immersive VR appears sufficient to adequately support spatial museum learning.

To our knowledge, our study was the first to examine the presence, usability, and enjoyment combined with cognitive and behavioral outcomes in a naturalistic, culturally significant context like a real museum exhibition.

This finding has important practical implications for educational applications of VR in museums, as it suggests that more affordable non-immersive desktop-based systems may be sufficient for content delivery and spatial learning, even though they provide less immersive experiences. An intriguing finding is that participants’ self-reported interest in exhibitions and cultural heritage did not significantly predict their outcomes from virtual museum experiences. This suggests that VR technology may have a democratizing effect, offering engaging experiences irrespective of previous cultural interests or museum attendance habits. This has positive implications for cultural outreach, as VR experiences could serve as effective tools for engaging audiences who might not typically visit traditional museums.

Our findings have several practical implications for cultural institutions considering VR implementations. First, museums should prioritize immersive technologies when seeking to maximize visitor engagement and satisfaction. However, the equivalent spatial learning outcomes for desktop-based VR indicate that less expensive technologies may be sufficient for educational objectives. The strong gender effects observed in our study suggest that VR museum experiences may be particularly effective for engaging female audiences but also highlight the need for design considerations that appeal to diverse user groups. Museum professionals and VR developers should consider how interface design, content presentation, and interaction modalities might be optimized to engage all demographic groups effectively. The finding that prior cultural interest did not predict VR experience outcomes suggests that virtual museums may be powerful tools for cultural outreach, potentially engaging audiences who might not otherwise visit traditional cultural institutions. This supports the post-pandemic trend toward hybrid cultural engagement models that blend physical and digital experiences [8,9].

Despite these innovative and practical contributions, this work is not without limitations. Including a sample of older adults would be important for analyzing how non-digital natives approach this technology and whether they benefit not only in experiential terms but also cognitively. Integrating metacognitive and cognitive measures with physiological measures, such as eye tracking, would help detect to identify museum elements that capture the most attention. Another limitation is the environmental complexity. As noted by Nori et al. [64], environmental complexity is one of the external factors that most influences participants’ visuospatial abilities. The virtual environment in our study was structurally simple, though rich in objects, and this may have created a ceiling effect for spatial learning performance in both conditions.

## 5. Conclusions

This study demonstrates that immersive VR technology can significantly enhance virtual museum experiences, with HMD-VR providing superior user engagement, satisfaction, and behavioral intentions compared to desktop-based alternatives. The gender effects observed across multiple measures highlight the importance of considering demographic variables, as well as other individual differences (such as cognitive styles and digital immigrants vs. digital natives) in future studies of VR cultural applications. While immersive technology clearly provides experiential advantages, the equivalent performance on spatial learning tasks suggests that different VR implementations may be optimal for different objectives within cultural institutions.

## Figures and Tables

**Table 1:** Research domain and principal findings on HMD-VR vs. non-immersive VR.

Research Domain	Principal Findings on HMD-VR vs. Non-Immersive VR
Spatial navigation	Mixed results: non-immersive VR outperformed HMD-VR (e.g., [29]) but sometimes no differences were found [30,35]
Educational learning	HMD-VR enhances engagement and long-term retention for learning by cultivating longer visual attention and fostering a higher sense of immersion, though students’ short-term retention remains the same across all conditions (e.g., [36,37,38])
Attention	Mixed results: HMD-VR is sometimes better, while other times it is equivalent to the non-immersive VR experience [32,33,34]

**Table 2:** Inter-correlations amongst demographic variables and dependent variables.

	Age	Gender	Level of Education	CUQ	CHARE
Pleasantness	−0.15	0.19	−0.15	−0.06	0.09
Perceived immersion	−0.14	0.26 *	−0.07	0.02	0.14
Intention to repeat similar experiences	−0.00	0.26 *	−0.09	−0.01	0.26 *
Learning Time	−0.28 **	−0.12	−0.18	−0.08	−0.16

* *p* < 0.05; ** *p* < 0.001.

**Table 3:** Summary of the mediation analyses.

Path	Effect	SE	BootLLCI	BootULCI
VR condition → pleasantness	7.18	2.99	1.23	13.12
VR condition → perceived immersion	9.67	3.38	2.93	16.40
pleasantness → learning time	0.05	0.03	−0.01	0.11
perceived immersion → learning time	−0.01	0.02	−0.06	0.04
VR condition → pleasantness →learning time (indirect effect)	0.39	0.28	−0.01	1.06
VR condition → perceived immersion → learning time (indirect effect)	−0.11	0.26	−0.68	0.37
VR condition → y (direct effect)	0.23	0.69	−1.15	1.60
R^2^=15				
F(8,78) = 1.69				

Note. *n* = 87. SE = Standard Error, LLCI = Lower Limit of the 95% Confidence Interval, ULCI = Upper Limit of the 95% Confidence Interval. All mediation analyses are performed controlling for age, gender, education, frequency of PC use, and CHARE.

## Data Availability

The data are available upon request to the authors.
